# Techniques for Obtaining High-quality Recordings in Electrocochleography

**DOI:** 10.3389/fnsys.2020.00018

**Published:** 2020-04-15

**Authors:** Michael J. Simpson, Skyler G. Jennings, Robert H. Margolis

**Affiliations:** ^1^Department of Communication Sciences and Disorders, University of Utah, Salt Lake City, UT, United States; ^2^Audiology Incorporated, Arden Hills, MN, United States

**Keywords:** electrocochleography, compound action potential (CAP), cochlear microphonic, tympanic membrane electrode, stimulus artifact

## Abstract

There are several technical challenges to obtaining high-quality recordings of cochlear potentials in human electrocochleography (ECochG). These challenges include electrical artifacts from devices such as acoustic transducers, biological artifacts from excessive myogenic and electroencephalographic potentials, and issues associated with the placement of a tympanic membrane (TM) electrode on the eardrum. This article presents approaches for dealing with these challenges for ECochG measurement using a TM electrode. Emphasis is placed on eliminating stimulus artifact, optimizing the placement of the electrode, and comparing a custom-made electrode with a commercially-available electrode. This comparison revealed that the custom-made electrode results in greater subject comfort, superior ease of placing the electrode on the eardrum, and larger compound action potential (CAP) amplitudes.

## Introduction

**Figure 1:**
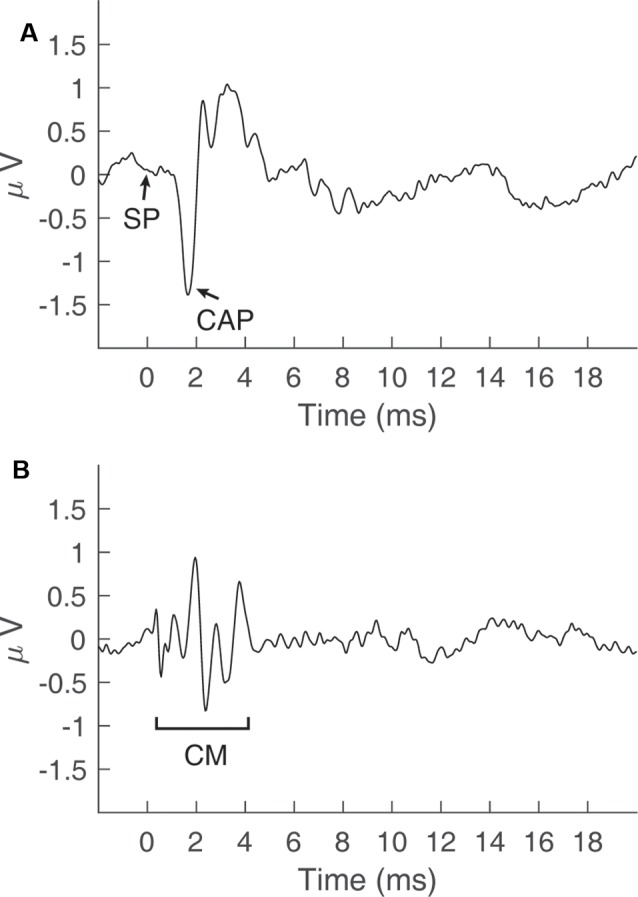
Cochlear potentials recorded *via* electrocochleography in a normal-hearing subject. **(A)** The summating potential (SP) and compound action potential (CAP) recorded by adding responses to a click stimulus in alternating polarity at 90 dB peSPL. **(B)** The cochlear microphonic (CM) potential obtained by taking the difference in responses to a click stimulus in alternating polarity at 90 dB peSPL.

Electrocochleography (ECochG) involves the measurement of stimulus-evoked potentials generated by cochlear hair cells and auditory nerve fibers (Eggermont, 2017). These potentials include cochlear responses to the onset and the steady-state portions of an acoustic stimulus. The onset response is generated by the synchronous firing of auditory nerve fibers and is known as the compound action potential (CAP, Figure 1A; Derbyshire and Davis, 1935). Steady-state responses include alternating current (AC), and direct current (DC) potentials. For high-frequency tones (i.e., >2000 Hz), the AC potential is generated primarily by the outer hair cells (OHCs) and is often referred to as the cochlear microphonic (CM, Figure 1B; Dallos, 1976). For low-frequency tones (e.g., 500 Hz), the AC potential is generated by a mixture of OHC activity and regular auditory nerve firing, or phase-locking, to the period of the tone. This phase-locking of the CAP to the period of a low-frequency tone has been referred to as the auditory nerve neurophonic (Snyder and Schreiner, 1984) and is related to the “auditory nerve overlapped waveform” (Lichtenhan et al., 2013). The DC component of steady-state ECochG responses is generated by a mixture of inner hair cells (IHCs) and OHCs, and is known as the summating potential (SP, Figure 1A; Durrant et al., 1998). Although the SP is visible in Figure 1A, this potential is often difficult to identify when elicited by transient stimuli (Ferraro et al., 1994). The SP is considered a steady-state potential and is best elicited in response to non-transient stimuli, such as tone bursts (Margolis et al., 1995).

Measurement of ECochG is based on principles of evoked-potential electroencephalography (EEG), where recordings are obtained by averaging the responses to repeated presentations of the acoustic stimulus. The primary distinction between ECochG and other auditory evoked potentials is the placement of an electrode on the cochlear promontory (Ruth and Lambert, 1989), tympanic membrane (TM; Cullen et al., 1972), or ear canal (Coats, 1974), rather than on the scalp. ECochG is often used in the clinical diagnosis of certain cochlear pathologies, such as Meniere’s disease (Hornibrook, 2017), and to assess cochlear sensitivity in laboratory animals (Liberman, 1978). In the clinic, Meniere’s disease is associated with an abnormally large SP/AP amplitude ratio (Gibson et al., 1977) and a larger-than-normal tone-burst SP (Margolis et al., 1995). This article is concerned with the technical challenges that must be addressed to obtain high-quality ECochG recordings in clinical or research laboratory settings. These challenges include eliminating electromagnetic artifacts, ensuring proper electrode placement, and promoting subject comfort/restfulness. Failure to address these technical challenges may result in contaminated electrophysiological responses and suboptimal signal-to-noise-ratios (SNRs) in ECochG recordings.

## Non-stimulus Related Electrical Artifact

Auditory evoked potentials (e.g., ECochG) are small compared to electromagnetic potentials produced by electrical outlets, overhead lights, and other electronic equipment. Electromagnetic radiation from these sources can contaminate electrophysiological recordings. Standard approaches to reduce electrical artifact are averaging, common-mode rejection, filtering, and shielding (Skoe and Kraus, 2010). Computer averaging increases the SNR between random electrical activity (noise) and deterministic signals (auditory evoked potentials), and this increase grows at a rate proportional to the square root of the number of sweeps. Common mode rejection relies on phase cancellation between the inverting and non-inverting electrodes *via* a differential amplifier. Signals that are common to the inverting and non-inverting electrodes are minimized, while differences are amplified. Frequently used ECochG electrode montages take advantage of common-mode rejection by placing one electrode near (e.g., ipsilateral TM electrode) and the other far (e.g., contralateral mastoid) from the presumed neural generator (e.g., ipsilateral auditory nerve). Electromagnetic radiation produced by outlets/lights is a 60 Hz sinusoid (i.e., line noise, 50 Hz in Europe) and can be filtered out of recordings by implementing notch filters at the line noise frequency and the corresponding harmonics. Additionally, reducing the recording window to an integer multiple of one half of a cycle of the line noise frequency [e.g., 1/(60*2)] can further attenuate line noise by adding this artifact in opposite polarity during averaging.

## Stimulus-Related Electrical Artifact

Presentation of an acoustic stimulus *via* headphones, earphones, or sound field transducers results in the generation of electromagnetic radiation near the transducer. This radiation is an electromagnetic copy of the acoustic stimulus and thus creates a “stimulus artifact.” To confirm the presence of a stimulus artifact, the tubing of an insert transducer can be crimped to reduce the level of the stimulus reaching the subject’s ear canal. The presence of an electromagnetic copy of the stimulus under such conditions is consistent with stimulus-related electrical artifact rather than a biologically-generated signal, such as the CM (Choudhury et al., 2012). Methods to manage stimulus artifact include, alternating the polarity of the stimulus (Aiken and Picton, 2008), using insert earphones, and magnetically shielding the transducer (Margolis, 1999). Additionally, a recent approach involves placing a pair of insert earphones adjacent to each other so their electric fields cancel during the simultaneous presentation of a stimulus in opposite polarity (Polonenko and Maddox, 2019). When measuring the CAP, stimulus artifact is reduced by averaging responses elicited by stimuli presented in alternating polarity; however, the CM is also eliminated in the process. Stimulus artifact is difficult to distinguish from the CM because the CM is an AC potential that mimics the presented stimulus (Adrian, 1931). For clicks, the use of insert earphones can temporally separate the CAP from the stimulus artifact. This is because the length of the transducer tubing creates a time delay between the artifact and the evoked response; however, this approach may not be successful for potentials evoked by longer stimuli, such as tone bursts (Margolis et al., 1995). Lastly, shielding and grounding the transducer and associated cabling eliminates stimulus artifact by providing an electrical drain for the electromagnetic radiation produced by the transducer. Through proper shielding, all cochlear evoked potentials (CAP, CM, SP) are preserved in recordings.

## Electrode Selection and Placement

Transtympanic, tympanic, and extratympanic electrode placements have been used in extracochlear ECochG recordings (Ruth and Lambert, 1989) in subjects with normal and impaired hearing. For hearing-impaired patients with cochlear implants, intracochlear EcochG can be conducted by assigning active and reference electrodes to contacts along the implant electrode array (Calloway et al., 2014; Campbell et al., 2015; Dalbert et al., 2018). Transtympanic placement involves inserting an electrode through the TM and onto the round window, or cochlear promontory (Portmann and Aran, 1971). This proximity to cochlear structures yields CAPs that are 5–10 times larger than extratympanic placements (Mori et al., 1982); however, transtympanic placement is not commonly used, as it is invasive and requires specialized training to administer (Ferraro and Krishnan, 1997). In clinical settings, ECochG is most commonly conducted using the less-invasive extratympanic/tympanic approaches, which involve placing an electrode in the ear canal, or on the TM. A common extratympanic electrode consists of a gold-foil covered foam insert, known as a “tip-trode” (Bauch and Olsen, 1990). Placing this electrode in the ear canal is associated with subject comfort, low cost, and ease of administration compared to tympanic and transtympanic approaches. Despite these advantages, CAP amplitudes in response to 90 dB nHL clicks produced by 100 μs pulses are three times larger when measured with a TM electrode compared to an ear canal electrode (Lilly and Black, 1989). Given that the TM electrode provides the largest SNR of non-invasive ECochG approaches, the remainder of the text will focus on ECochG measured with tympanic electrode placement. A comparison of CAP amplitudes for extratympanic, tympanic, and transtympanic electrode placements was quantified in Figure 1 of Margolis (1999).

Margolis et al. (1995) reported that placement of a TM electrode on the umbo provides the largest SNR compared to other TM or tympanic annulus locations. However, placement on the umbo is often difficult due to poor visualization of the TM while the electrode is *in situ*. Ideally, TM electrode placement is conducted with a microscope by a skilled expert (Margolis et al., 1995). In the absence of a microscope, the common practice is to ask the subject to verify that the electrode has reached the eardrum. This is done by advancing the electrode toward the eardrum while the subject reports changes in the ear canal/TM sensation. A disadvantage of this approach is that subjects are particularly sensitive to pressure placed on the bony portion of the ear canal. A subject may mistake this pressure for the contact between the electrode and the TM. Margolis (1999) compared ECochG responses for TM electrode placements under direct visualization *via* a surgical microscope, vs. no visualization, and reported that visualization provides a 1.54 μV increase in CAP amplitudes in response to a ~90-dB nHL click. This result suggests that subject report is a poor indicator of optimal electrode placement. An alternative method to solely relying on subject report involves visualizing the ear canal after TM electrode removal to determine the specific site of contact. This is done by searching for an area of redness and residual electrode gel left by the TM electrode on the eardrum (Smith et al., 2016). Unfortunately, this method of visualization is only possible after testing has been completed.

### Subject-related Challenges

The restfulness of the subject can have an impact on the quality of ECochG recordings, as restful subjects tend to have quieter background EEG activity (Eggermont and Odenthal, 1974). Testing often lasts an hour or more. If a restless subject frequently shifts positions during the session, the myogenic artifact will contaminate recordings and result in a poor SNR. Muscle movements can also shift the position of the transducer and/or electrode, leading to changes in electrode impedance and ear canal sound pressure level (SPL) throughout the experiment. The temperature of the room, too hot or too cold, can affect subject relaxation and result in sweating which may alter scalp electrode impedances (Luck, 2014). Response averaging and artifact rejection methods can eliminate some unwanted muscle potentials from subject unrest; however, the effects of excessive subject movement on recording quality cannot be rectified without reinstructing the subject and making the recording environment more comfortable. If possible, the subject should be given the option to request a pause in testing before any major shifts in position are made.

Another subject-related challenge stems from the post-auricular muscle (PAM) artifact which is elicited by acoustic stimulation and may appear as a large potential 12–16 ms after stimulus presentation (Yoshie and Okudaira, 1969). Poor neck support, subject anxiety, and turning the eyes toward the ear receiving the stimulus can increase this artifact (Mahendran et al., 2007). A workaround for the PAM artifact is to shorten the recording window to less than 12 ms when using appropriately brief stimuli. Providing the subject with a supportive neck pillow can decrease this artifact by reducing neck tension. Lastly, Hall (2007) recommended placement of the reference electrode on the nape of the neck, as opposed to the contralateral mastoid, in order to reduce PAM artifact.

## Materials and Methods

Below are solutions to address the technical challenges of ECochG measurements, as implemented at the University of Utah. Central to these solutions is the development and testing of electromagnetically-shielded transducers and a custom-made TM electrode. Materials and methods used in this study were approved by the institutional review board (IRB) at the University of Utah.

## Managing Stimulus-Related Electrical Artifacts

**Figure 2:**
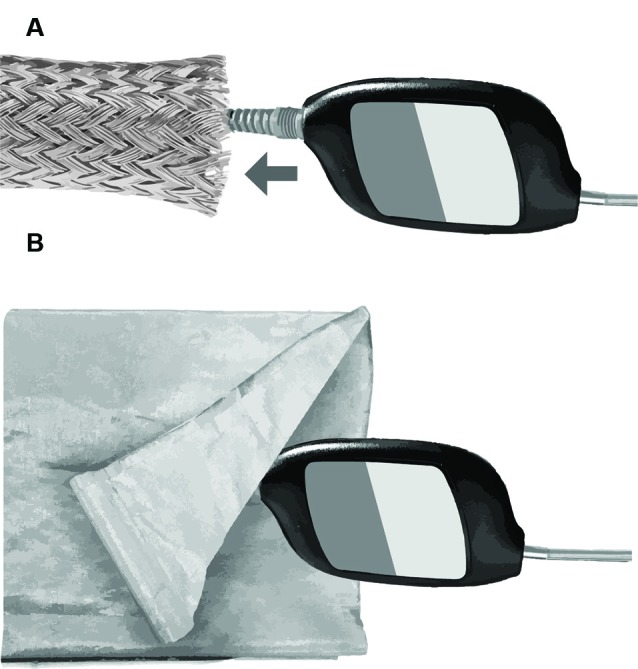
Methods for electromagnetic shielding without altering the headphone transducer and cabling. **(A)** Braided copper sleeve. **(B)** Faraday fabric.

Several approaches were tested for eliminating stimulus artifacts *via* shielding the transducer. The objective was to develop a Faraday cage around the transducer to drain stimulus artifact to earth ground. An initial approach involved a braided, expandable, tinned-copper sleeve (Electriduct, Pompano Beach, FL, United States), which was pulled over the transducer body and cable and connected to earth ground (Figure 2A). The sleeve successfully shielded the transducer; however, it was heavy and not insulated, thus posing a potential electric shock hazard to subjects. The use of a lighter, copper/nickel “Faraday fabric” (MOS Equipment, Santa Barbara, CA, United States) eliminated the problems associated with the weight of the tinned-copper sleeve; however, the shielding effectiveness of this fabric was inconsistent and posed a similar electric shock risk (Figure 2B).

**Figure 3:**
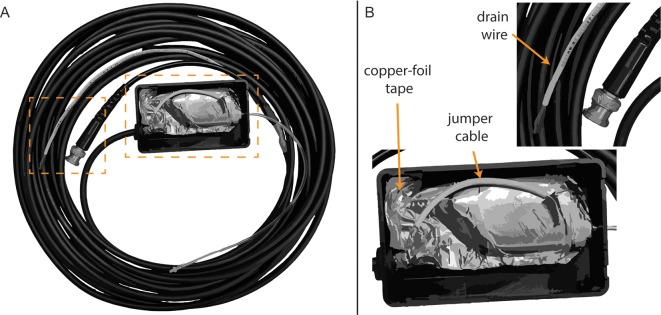
Modified earphone transducer and cabling. **(A)** Full view of modified transducer and cabling. **(B)** Close-up view of foil shielding, jumper cable, and drain wire.

Campbell et al. (2012) described a method of electromagnetic shielding that involved covering the transducer and cabling with copper foil, followed by an insulating layer of electrical tape. This method requires no modification of the transducer; however, the shielding and tape adds extra bulk, is subject to wear, and is difficult to remove. The shielding method used at the University of Utah involves modifying the insert earphones (Figure 3), based on an approach developed by Eric Fournier and Robert Margolis at the University of Minnesota (personal communication). First, copper-foil tape (3M, Maplewood, MN, United States) was used to tightly cover the body of ER-3C earphones. The metal nipple used to connect the transducer tubing was left exposed. Next, a small portion of the stock cable, including the plug that inserts into the transducer body was retained and the remaining stock cable was discarded and replaced with a shielded twin-lead cable (Mogami, Torrence, CA, USA). The rubber insulation was stripped from one end of the twin-lead cable to expose the leads and shielding. Fibers from the cable shielding were twisted together and soldered to a jumper cable, which was soldered to the copper-foil tape surrounding the transducer (Figure 3B). Positive and negative leads from the retained portion of the stock cable were stripped and soldered to corresponding leads of the twin-lead cable. The stock cable was then plugged into the transducer body and copper-foil tape was used to cover the stock cable and the exposed part of the twin-lead cable. Next, the shielded transducer was housed inside of a 4” × 2.5” × 1” plastic junction case (Pinfox, China) with custom-drilled holes to accommodate the transducer tubing and cable. A rubber grommet was used to secure the twin-lead cable at the entrance to the plastic case. The insulation of the non-transducer end of the twin-lead cable was stripped and the cable leads were soldered to a BNC connector, which was used to connect the transducer to signal processing hardware. Lastly, the cable shielding of the non-transducer end of the twin-lead cable was soldered to a drain wire (Figure 3B), which was connected to earth ground by a faceplate screw of an electrical outlet. Proper electromagnetic shielding was verified on human participants by clamping the transducer tubing and measuring ECochG under conditions where the transducer shielding was, or was not connected to earth ground. Clamping the transducer ensures the subject does not perceive the acoustic stimulus and thereby minimizes the CM. The stimulus was a 75 ms, 1,000 Hz tone burst presented at 95 dB SPL. Recordings from our laboratory often include a probe microphone to measure ear canal SPL simultaneously with ECochG. This microphone can produce stimulus-related artifact and was shielded using similar methods as the insert earphones. This microphone was not used during measurements obtained to compare TM electrodes.

## Placement of TM Electrodes

**Figure 4:**
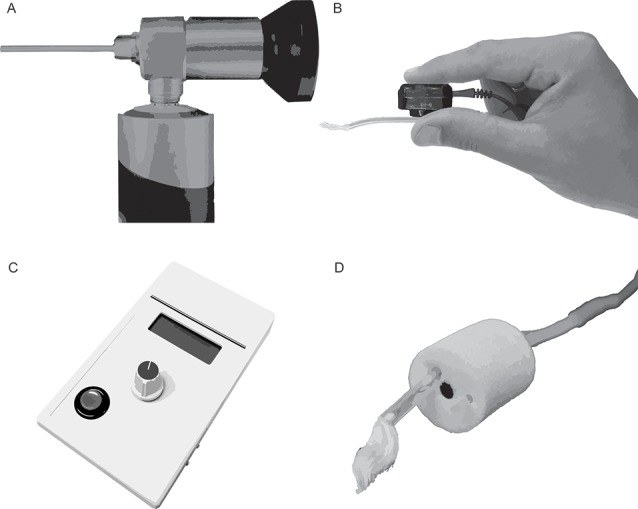
Toolsfor improving the placement of extratympanic electrodes on the tympanic membrane (TM). **(A)** Otoendoscope. **(B)** Bone oscillating headset coupled to a TM electrode. **(C)** Impedance meter. **(D)** Foam insert with TM electrode threaded through a custom-drilled bore.

Several approaches were evaluated qualitatively to optimize placement of the TM electrode on the eardrum and improve subject comfort relative to standard clinical ECochG procedures for TM electrodes. After preparing the electrode with a conductive gel or a soak in saline solution, standard clinical procedures consist of advancing the electrode into the ear canal until the patient perceives the electrode has reached the TM. Patients describe this perception as a pressure or tickling sensation. Some patients report hearing a change in auditory perception when the electrode touches the eardrum. With the electrode *in situ*, the clinician then places a foam insert in the ear canal. This insert couples the earphones to the patient’s ear canal and serves to retain the position of the TM electrode on the eardrum. Approaches described here were evaluated relative to these standard procedures and include the use of an otologic endoscope, a bone oscillator, an impedance meter, and a bored foam insert (Figure 4). Although these approaches were helpful, the use of a custom-made electrode, described in a later section, was superior to these approaches in terms of ease of administration, CAP amplitudes, and subject comfort.

• Visualizing the eardrum using standard otoscopes can be difficult when a TM electrode occupies the ear canal. An otologic endoscope (Figure 4A; Olympus Medical, Center Valley, PA, USA) provided better visualization of the contact between the electrode and the TM compared to standard otoscopes due to its small telescope diameter and a wide-angle lens. The head of a TM electrode often consists of a cotton wick or bulbous, gel-covered conductor. The electrode head fills a significant portion of the ear canal. An otoendoscope allows the experimenter to view around the electrode head and adjust the electrode’s position, as necessary.
• A standard bone-oscillating headset (Figure 4B) was used to differentiate between electrode placement on the eardrum from placement on the ear canal wall. The experimenter delivers a continuous 100-Hz tone and pinches the distal end of the TM electrode to the bone oscillator while the head of the electrode is advanced into the ear canal. This approach couples the vibration of the oscillator to the shaft and head of the electrode. Subjects report an increase in the loudness of the 100-Hz tone when the electrode touches the TM compared to the ear canal wall. Subjects reported that higher tone frequencies (250, 500, 1,000 Hz) did not produce as large of an increase in loudness, compared to 100 Hz, when the electrode contacted the TM.
• An impedance meter (Figure 4C; Bio-Medical, Clinton Township, MI, USA) with a large dynamic range (0–200 kΩ) and high-resolution step size (100 Ω) typically registers an appreciable increase in impedance when the electrode contacts the TM rather than the ear canal wall (Margolis et al., 1995). Despite this, the size and direction of this change in impedance varies widely among subjects, based on our clinical and laboratory impressions.
• Similar to the vented earmold design of Durrant (1990), a bore (Figure 4D) was created using a 1.65 mm core drill (OakTree, Chesterfield, MO, USA) through a standard adult-size foam insert to serve as a pathway for the TM electrode. The head of the electrode is threaded through the bore before preparing the electrode head with a conductive gel or saline. The foam insert is then placed in the ear canal with the electrode head extending slightly past the medial end of the foam insert. After placing the foam insert, the TM electrode is advanced slowly until the subject reports that the electrode has reached the eardrum. This approach improves subject comfort compared to methods that place the foam insert with the electrode *in situ*. Moreover, this approach allowed for fine-tuned advancement of the electrode, as careful selection of the foam bore diameter resulted in a friction fit with the electrode shaft. Despite these benefits, this approach relies on subject report rather than direct visualization to confirm the electrode has reached the eardrum.

Of the approaches above (otologic endoscope, bone oscillator, impedance meter, bored insert), our laboratory has adopted the bored insert approach when using commercially-available electrodes, as this approach is easy to administer and well tolerated by our subjects.

## Development and Testing of a Custom-made Electrode

### Electrode Design and Production

**Figure 5:**
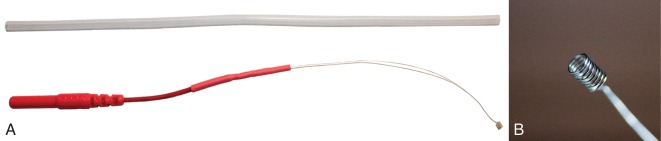
University of Utah (U of U) TM electrode. **(A)** A full view of the U of U TM electrode next to a segment of Silastic tubing. The tubing diameter (2 mm) is equal to that of many commercial TM electrodes. **(B)** Close-up view of the coiled end of the U of U TM electrode.

An alternative extratympanic electrode [University of Utah (U of U) TM electrode] was developed to improve electrode placement and subject comfort relative to the approaches discussed above. This electrode has a lower profile and is less rigid compared to commercially-available electrodes (Figure 5). The design consists of a 6”, 0.1397 mm diameter, Teflon-coated silver wire attached to a touch-proof connector. Roughly, 1.25” of the Teflon coating is removed from both ends of the 6” wire using fine-point tweezers or wire strippers (Model 1004, Klein Tools, Chicago, IL, USA). One end of the silver wire is wrapped around the leads of a 1.5 mm female touch-proof connector (Plastics One Inc., Roanoke, VA, United States), and heat shrink tubing is used to secure and insulate the connection. The opposite end of the wire is wrapped around a 1 mm diameter rod (metal reamer from a 1.65 mm bore drill, OakTree, Chesterfield, MO, USA) to form a small coil that serves as the electrode head (Figure 4B). ECochG recordings were compared between the U of U TM electrode and a commercially-available electrode (Lilly TM-Wick Electrode, Intelligent Hearing Systems, Miami, FL, USA).

## Subjects

Nine young adults (mean age = 24 years, eight females) with normal hearing were recruited for the experiment. Hearing status was defined as pure-tone air conduction thresholds <20 dB HL at audiometric frequencies from 250–8,000 Hz. All subjects had type-A tympanograms, consistent with normal middle ear function.

## Stimuli

The stimulus was a click produced by a 100 μs electrical pulse delivered to the insert earphones and presented from 40 dB peak-equivalent SPL (peSPL) to 90 dB peSPL, in 10 dB steps in descending order. Clicks were presented in alternating polarity for 1,024 recording sweeps.

## Equipment and Procedures

Recordings were obtained using Tucker Davis Technologies (TDT, Alachua, FL, USA) hardware and software. The hardware consisted of a high-performance workstation (TDT-WS4), multi-input/output signal processor (TDT-RZ6), low-impedance headstage (TDT-RA4LI), bio amplifier (TDT-RA4PA), and electromagnetically-shielded insert earphones (ER-3C, Etymotic Research, Elk Grove, IL, USA). Evoked potentials were recorded using TDT Synapse software, which was controlled *via* custom Matlab (The Mathworks, Natick, MA, USA) code and the Synapse Application Program Interface (API).

The active electrode was located on the TM, while the ground and reference electrodes were located on the lower forehead and the ipsilateral earlobe, respectively. This electrode montage was adopted based on the findings of Kumaragamage et al. (2015), who reported increased SNRs when the reference electrode was placed on the ipsilateral ear, as opposed to a contralateral mastoid/earlobe placement. This montage decreases the distance between active and reference electrodes, resulting in increased common-mode rejection of physiologic artifacts from muscle and non-stimulus evoked brain activity. Before testing, the ear canal was inspected for the presence of cerumen that may interfere with advancing the TM electrode and placing the foam insert. If needed, the cerumen was removed using a curette or irrigation. The ear canal was hydrated and drained with saline before placing the TM electrode. Testing with the U of U TM electrode was conducted first, followed by testing with the commercially-available electrode in all nine subjects. To test for order effects, the same procedure was repeated in a follow-up experiment; except the commercially-available electrode was tested before the U of U TM electrode. Four of the original nine subjects were available to participate in the follow-up experiment. For testing with both electrodes, (1) impedances were actively monitored during electrode placement and subsequent introduction of the foam insert, (2) the proximal portion of the electrode was taped to the subject’s cheek for retention, and (3) the electrode was connected to the bioamplifier *via* a shielded cable (Intelligent Hearing Systems, Miami, FL, United States), which connects the shielding to the ground electrode with a jumper cable.

## Placement of the Commercially-Available Electrode

The commercially-available electrode was placed on the TM using the “bored-insert” method described above. Specifically, the electrode was removed from packaging and threaded through the hand-drilled bore of the foam insert before the cotton wick was soaked in saline for at least 10 min. Sound pressure measurements revealed that this alteration of the foam insert had no effect on the peSPL of 100 μs pulses measured in a coupler, or the ear canal of subjects. After soaking in saline, the electrode head was retracted until it just passed the ear canal side of the insert. After the insert expanded in the ear canal, the electrode was slowly advanced until the subject perceived the electrode touching the eardrum. Subjects were instructed to indicate when they felt pressure or a tickle sensation, or they heard something that sounded like an object touching their eardrum.

## Placement of the U of U TM Electrode

**Figure 6:**
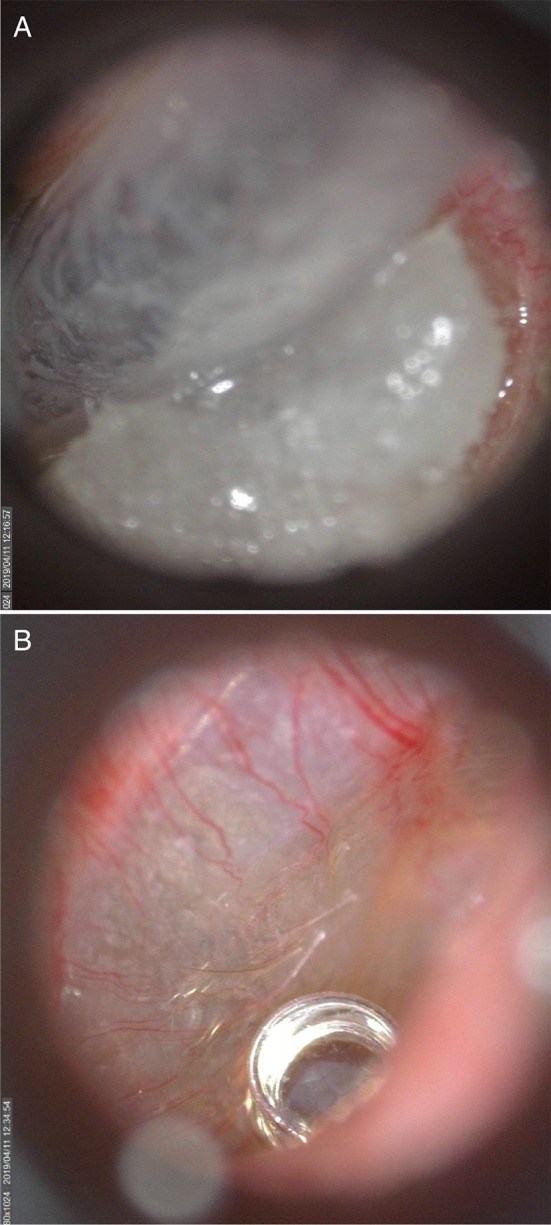
A commercially-available TM electrode in the ear canal **(A)** and Video otoscopic view of the University of Utah (U of U) TM electrode **(B)**.

The coiled end of the U of U TM electrode was coated with conductive gel (Spectra360, Parker Laboratories INC., Fairfield, NJ, USA) immediately before insertion into the ear canal. Otoscopy with a standard otoscope was conducted while placing the U of U TM electrode to visualize contact between the head of the electrode and the TM. Rubber tipped forceps were used to make fine adjustments of the wire to achieve placement near the umbo. These adjustments were made possible due to improved visualization of the electrode head in the ear canal (Figure 6B) compared to commercially-available electrodes (Figure 6A). Often normal-hearing subjects hear when the electrode makes contact with the TM. For ear canals with sharp bends and/or excess hair, it was helpful to straighten the ear canal with an endaural speculum (Oaktree Products, Chesterfield, MO, USA) to prevent the electrode from catching and bending on the walls of the ear canal. Although the U of U TM electrode was associated with higher impedances than the commercially-available electrode, this increase did not result in smaller CAP amplitudes or higher noise floors, as discussed below. The thin diameter and malleability of the U of U TM electrode ensured that subsequent placement of the foam insert did not result in discomfort to the subject.

## Data Analysis

CAP peak-to-peak amplitudes were detected and measured by a computer algorithm implemented in Matlab. The algorithm found the minimum (i.e., negative peak) in a temporal window extending 8 ms from the onset of the acoustic stimulus. The subsequent positive peak was found by computing the derivative for the remaining data points within the temporal window. The inflection point in which the derivative changed from positive to negative marked the positive peak. The peak-to-peak amplitude was measured as the difference in voltage between the initial negative peak and the subsequent positive peak. These measurements were verified by the first and second authors. The first author is a graduate student and Doctor of Audiology Extern with >200 h of clinical and research experience in ECochG, and the second is a clinically-trained audiologist and hearing scientist. In instances where the computer algorithm was judged to incorrectly identify the CAP, the experimenters manually measured and recorded the CAP using a custom graphical user interface. This interface presented the experimenters with recordings from each subject, one by one. Recordings from the highest click level appeared first, along with the associated computer-identified CAP. The experimenter (1) accepted the CAP measurement, (2) manually measured the CAP, or (3) indicated that the CAP was absent. This process was repeated for each click level, in descending order. The experimenters paid particular attention to the latencies of the computer-identified CAPs, as these latencies were expected to increase with decreasing click levels. The algorithm identified the correct CAP amplitude with 78% accuracy. Of the remaining 22% of incorrectly identified CAP amplitudes, 50% were associated with the two lowest levels (40 and 50 dB peSPL). The effects of electrode type (commercially available, U of U) and order (follow-up experiment) were evaluated using paired t-tests after correcting for multiple comparisons using Bonferroni’s method.

## Results and Discussion

### Electromagnetic Shielding

**Figure 7:**
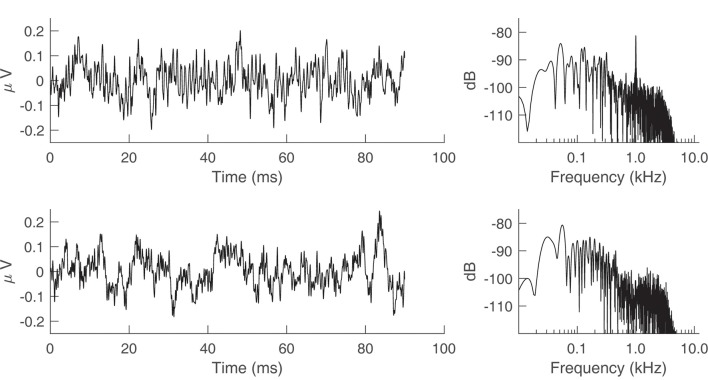
Electrocochleography (ECochG) recordings from a representative human subject with the transducer tubing clamped. The stimulus was a 75 ms, 1,000 Hz tone burst presented at 95 dB sound pressure level (SPL). (Top row) Time waveform and spectrum of the ECochG recording when the transducer is not grounded to the earth. (Bottom row) Time waveform and spectrum after earth grounding.

Figure 7 shows the time waveform and spectrum of an ECochG recording for a 75 ms, 1,000 Hz tone burst in a representative subject. The left and right columns of the top panel of Figure 7 are the results before the transducer shielding was grounded. A 1,000-Hz component is observed in the spectrum (top row, right column), consistent with the 1-ms periodic oscillations seen in the waveform (top row, left column). The bottom row of Figure 7 shows the result of connecting the transducer to earth ground, whereby the 1,000-Hz component is eliminated.

### Comparison of TM Electrodes

**Figure 8:**
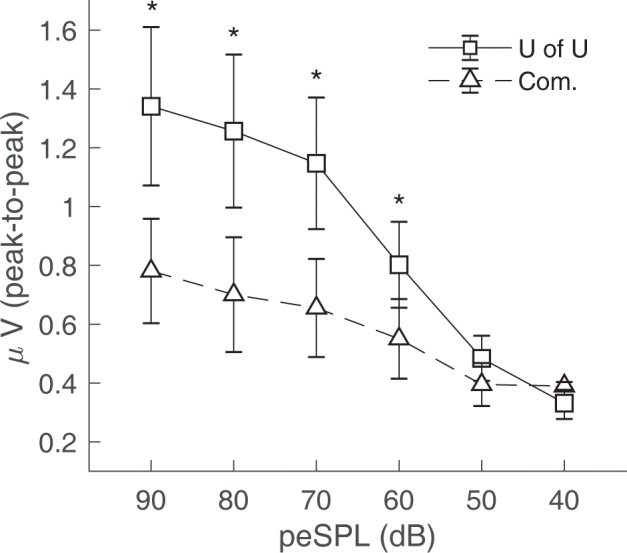
Peak-to-peak CAP amplitudes for the University of Utah (U of U) TM electrode and a commercially (Com.) available TM electrode. CAPs were measured in response to alternating polarity clicks in a descending intensity series from 90 dB peSPL to 40 dB peSPL. Asterisks indicate significance at the α = 0.05 level after Bonferroni correction. Error bars represent one standard error of the mean.

**Figure 9:**
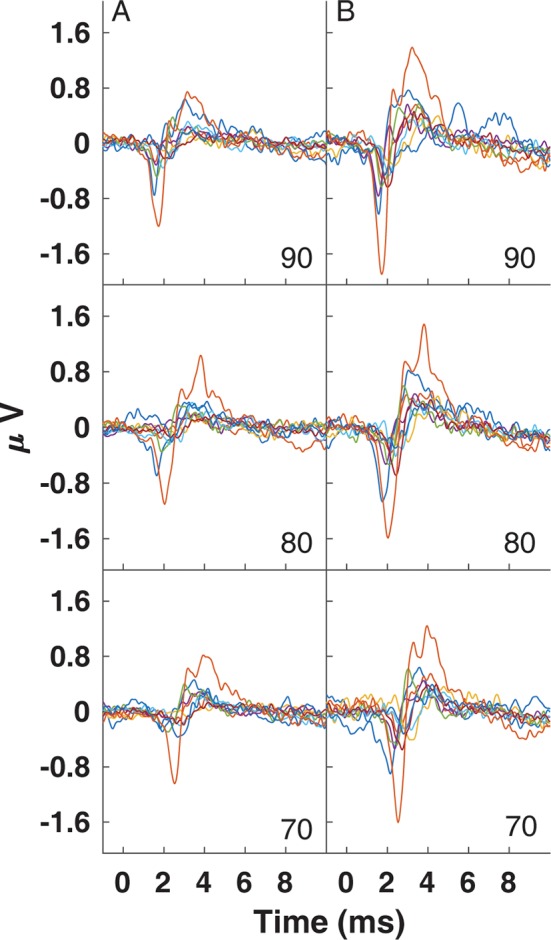
Traces for CAPs in individual subjects measured using a commercially-available electrode **(A)** and the University of Utah TM electrode **(B)** for clicks presented at 90 (top), 80 (middle) and 70 dB peSPL (bottom).

**Figure 10:**
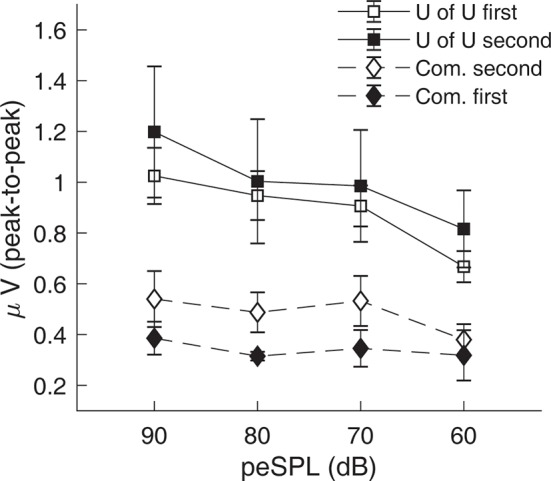
Results from the follow-up experiment showing that CAP amplitudes measured with the University of Utah (U of U) TM electrode are larger than those measured with a commercially-available electrode (Com.), regardless of the test order of the electrodes. Open symbols are data from the original order (U of U first, Com. second), while closed symbols are for the reverse order (Com. first, U of U second). Error bars represent one standard error of the mean.

CAP amplitudes for the original study in nine subjects are shown in Figure 8. CAPs were present for all subjects for clicks presented at 60 dB peSPL and above, except one subject had absent CAPs at 60 dB peSPL when responses were measured with the commercially-available electrode. For 50 dB peSPL clicks, CAPs were present for 8/9 and 7/9 subjects for recordings with the U of U TM electrode and commercially-available electrode, respectively. For 40 dB peSPL, these numbers were 5/9 and 3/9. Traces showing the average of alternating polarity sweeps for all subjects are shown as a function of level (rows) and electrode type (columns) in Figure 9. The U of U electrode yielded peak-to-peak CAP amplitudes that were 1.89–2.04 times larger in a given subject than those measured with the commercially-available electrode for clicks presented at 60, 70, 80, and 90 dB peSPL (*p* < 0.002 for all comparisons). Results from the follow-up experiment (Figure 10) in four returning subjects revealed that these larger amplitudes did not depend on the testing order of the electrodes (i.e., U of U electrode first or last) for CAP amplitude differences computed between electrodes for all click levels (*p > 0.05* for all comparisons). All subjects reported that the U of U TM electrode was more comfortable during placement and throughout testing.

This study reviewed several techniques for achieving high-quality ECochG recordings. Emphasis was placed on reducing stimulus-related artifact by shielding and grounding insert earphones, evaluating approaches to placing TM electrodes on the eardrum, and developing and testing a custom-made TM electrode. Results show that stimulus-related artifact is eliminated when transducer shielding is connected to earth ground; thus, enabling the recording of biological AC potentials, such as the CM, without contamination of electromagnetic artifacts. Our laboratory has adopted the bored-insert approach for placing commercially-available electrodes on the eardrum (Figure 4D), after evaluating alternatives such as placement using an otoendoscope, bone oscillator, or impedance meter. This approach allows the experimenter to advance the electrode toward the eardrum using fine adjustments due to the friction fit between the foam insert and the electrode shaft. Our participants reported that this approach is more comfortable than placing the foam insert after positioning the electrode, where subsequent insertion of the foam insert may transfer force to the TM electrode and result in increased pressure on the eardrum. A disadvantage of the bored-insert method is the inability to visualize the TM electrode during placement on the eardrum. This disadvantage led to the development of the U of U TM electrode. CAPs evoked by a 90 dB peSPL, 100 μs electrical pulse had higher amplitudes when measured with the U of U TM electrode compared to a commercially-available electrode, and this difference was independent of testing order. Similarly, the superior comfort of the U of U electrode did not depend on the order of testing, as reported by study participants.

Previous studies have measured CAPs in response to click stimuli using TM electrodes that terminate in a cotton wick or a small piece of foam, similar to the Lilly TM-Wick electrode used in this study. Often these electrodes are made in the laboratory (e.g., Ferraro and Durrant, 2006); however, a limited number of studies report purchasing and using the TM-Wick electrode to measure cochlear potentials. For 90 dB nHL clicks, baseline-to-peak amplitudes approach 1 μV in studies that used the TM-Wick electrode (Bonucci and Hyppolito, 2009; Lake and Stuart, 2019). The highest click level used in the current study was 90 dB peSPL, which is 32–35 dB lower than 90 dB nHL. This difference in level explains why the average baseline-to-peak amplitude (0.44 μV) measured using the TM-Wick electrode in this study did not reach the amplitudes reported in previous studies. In addition to the cotton wick-type electrode, another commercially-available TM electrode consists of an insulated silver wire whose tip is coated with a conductive hydrogel (Sanibel Supply, Eden Prairie, MN, USA). Procedures for measuring cochlear potentials with the Sanibel TM electrode are very similar to those used for the TM-Wick electrode. CAP amplitudes measured in response to clicks presented between 80–105 dB peSPL have been reported for several studies that used the Sanibel electrode (Stamper and Johnson, 2015; Harris et al., 2018; McClaskey et al., 2018), and are consistent with the 0.44 μV baseline-to-peak CAP amplitudes of the current study for clicks presented at 90 dB peSPL. These results show that the CAP amplitudes reported for the commercially-available electrode in the current study are consistent with CAP amplitudes obtained in previous studies that used a comparable commercially-available TM electrode.

The larger CAP amplitudes obtained with the U of U electrode, compared to the commercially-available electrode, are likely due to improved visibility of the eardrum while placing the electrode. This improved visibility allows the experimenter to more accurately place the electrode on or near the umbo, rather than on the ear canal wall or tympanic annulus. Singh et al. (1980) measured CAP amplitudes in response to 100 μs clicks presented at 80 dB nHL for TM electrodes positioned at several locations on the tympanic annulus. They found that an electrode positioned at 7 o’clock, relative to the axis of the malleus, resulted in a roughly 0.5 μV increase in CAP amplitude compared to electrodes positioned at 5:30 or 11 o’clock. Furthermore, Margolis et al. (1995) reported substantially larger CAP amplitudes evoked by 100 μs clicks compared to previous studies that used similar levels (88 dB nHL) and attributed these larger amplitudes to the placement of the electrode on the umbo under direct microscopic visualization. Our findings are consistent with these studies and suggest that the placement of the TM electrode on or near the umbo under direct visualization contributes substantially to obtaining robust CAP amplitudes.

The preliminary results of the U of U TM electrode are limited to normal-hearing adults tested in a laboratory setting. At this time, it is unclear how this electrode performs in a clinical setting in patients with cochlear hearing loss from noise exposure, ototoxicity, aging, or Meniere’s disease. Currently, the U of U TM electrode is not commercially available, and is only recommended for use in a laboratory setting after IRB approval has been granted. The electrode is not difficult to construct for a researcher or lab assistant who is comfortable stripping insulation from wires and applying shrink wrap with a heat gun. A trained research assistant can readily make the electrode in the laboratory in about 7 mins for under $5 per electrode, excluding the cost of labor. Other advantages include an improved level of comfort reported by subjects during placement and throughout testing. This is likely due to the decreased mass and stiffness of the electrode compared to the silicone-tube design present in most commercial TM electrodes.

## Summary and Conclusions

This article outlined techniques to improve the quality of ECochG recordings in clinical and research settings. The major challenges to recording high-quality ECochG are contamination by artifacts (electrical, stimulus-related, myogenic), and optimal placement of the TM electrode. Stimulus artifact can be eliminated by using shielded transducers with shielding connected to earth ground. Subject report is a poor method for verifying placement of the TM electrode on the eardrum, whereas placement under direct visualization is expected to produce relatively larger CAP amplitudes. For commercially-available electrodes, subject comfort can be improved by advancing the TM electrode through a foam insert modified with a custom-sized bore. Compared to commercially-available electrodes, the U of U TM electrode is associated with improved visualization of electrode placement, greater subject comfort, and larger CAP amplitudes.

## Data Availability Statement

The datasets generated for this study are available on request to the corresponding author.

## Ethics Statement

The studies involving human participants were reviewed and approved by The University of Utah Institutional Review Board. The patients/participants provided their written informed consent to participate in this study.

## Author Contributions

MS and SJ wrote the manuscript, designed the experiments, and developed the tympanic membrane electrode. MS collected the data. RM consulted on techniques for eliminating stimulus artifact and edited the manuscript.

## Conflict of Interest

RM was employed by the company Audiology Incorporated. The remaining authors declare that the research was conducted in the absence of any commercial or financial relationships that could be construed as a potential conflict of interest.
